# An Innovative Approach to Manganese-Substituted Hydroxyapatite Coating on Zinc Oxide–Coated 316L SS for Implant Application

**DOI:** 10.3390/ijms19082340

**Published:** 2018-08-09

**Authors:** Karuppasamy Prem Ananth, Jinxing Sun, Jiaming Bai

**Affiliations:** 1 Shenzhen Key Laboratory for Additive Manufacturing of High-Performance Materials, Shenzhen 518055, China; sjx476526525@163.com; 2 Department of Mechanical and Energy Engineering, Southern University of Science and Technology, Shenzhen 518055, China

**Keywords:** hydroxyapatite, corrosion zinc oxide, electrodeposition, bilayer coating, biomedical implant

## Abstract

In this paper, the synthesis of porous manganese substituted hydroxyapatite (Mn-HAp) coating on zinc oxide (ZnO) coated stainless steel (316L SS) using the electrodeposition technique is reported. The structural, functional, morphological, and elemental analyses are characterized by various analytical techniques including X-ray diffraction (XRD), Fourier transform infrared spectroscopy (FT-IR), field emission scanning electron microscopy (FE-SEM), transmission electron microscopy (TEM), and X-ray photoelectron spectroscopy (XPS). Results of electrochemical techniques such as cyclic polarization and impedance show that the Mn-HAp coating on ZnO coated 316L SS has the highest corrosion resistance in simulated body fluid (SBF) solution. Moreover, dissolution of metal ions was extremely reduced, as evaluated by inductively coupled plasma-atomic emission spectroscopy (ICP-AES). The adhesion and hardness of Mn-HAp/ZnO bilayer coatings have superior mechanical properties over individual coatings. Further, the biocompatibility of in vitro osteoblast attachment, cell viability, and live/dead assessment also confirmed the suitability of Mn-HAp/ZnO bilayer coating on 316L SS for orthopedic applications.

## 1. Introduction

Metals have been used for implant applications since 1895. Currently, stainless steel (316L SS) is the most used alloy in orthopedic and dental implant applications, owing to its high corrosion protection, excellent mechanical strength, good processsability, biocompatibility, and low cost [1]. This material is employed in load-bearing applications such as bone fixation and total joint replacement in the human body [2]. However, the constant involvement of its corrosive nature and biocompatibility in physiological mediums is significant, since a corrosive metal can be very harmful to the human body, and 316L SS metal might create localized corrosion in the body [3]. In some cases, metallic ions released from 316L SS, such as iron, chromium, and nickel, can accumulate in neighboring tissues, and local systemic effects could affect their proliferation and differentiation [4]. Therefore, in order to overcome the adverse reactions in the human body and to increase the lifespan of implanted orthopedic devices, surface treatment of metals is often required.

Coating the surfaces of implant devices with an organic self-assembled monolayer [5], glass [6], or ceramic is an effective method of corrosion protection [7,8]. At present, the focus is on zinc oxide (ZnO) coating, which has attracted interest due to its excellent anticorrosion properties and potential in implant applications [9]. Since ZnO nanoparticles are nontoxic, they can be used to produce environmentally friendly coatings, and they also have excellent optical, chemical, mechanical, and biological properties [10,11]. Several attempts have been made to use nanosized (small size and high surface area) particle coatings on implant devices. As mentioned above, such coatings would be corrosion protective, a significant advantage [12,13].

Calcium phosphate (CaP)–based bioceramics, particularly hydroxyapatite (HAp), are basic inorganic components for hard biological tissues such as bones and teeth due to their close resemblance to the mineral phase and crystalline structure [14,15]. The application of bioactive calcium phosphate (Cap) coatings could supply increased amounts of Ca^2+^ and PO_4_^3−^ in initial stages of implantation, and thus the transformation to a smaller amount of soluble biocompatible hydroxyapatite can be achieved [16]. HAp has two crystal forms: (i) monoclinic, space group P2_1_/b, and (ii) hexagonal, space group P6_3_/m. Only the hexagonal phase is of practical significance, because the monoclinic form is weakened by the presence of even small amounts of foreign ions [17]. Normally, human bone contains trace amounts of minerals such as sodium (Na+), magnesium (Mg^2+^), strontium (Sr^2+^), zinc (Zn^2+^), silicon (Si^4+^), and manganese (Mn^2+^) [18,19]. The substitution of ions in such species is considered to have a significant influence on the physical, chemical, and physiological properties of solid bones and teeth and subsequently on the mineralization, demineralization, and remineralization process of calcified tissues [20,21]. Among the various ion substitutions, manganese (Mn^2+^) substituted HAp significantly improves the superiority of bone repair in biotechnological coatings [22]. Hence, incorporating manganese into the apatite structure is of great interest because of its improvement in mechanical properties, controlled cell interactions with the extracellular matrix, and activation of cellular adhesion [23]. Many coating techniques have been applied to improve the corrosion resistance of 316L SS in physiological fluids, among which plasma spraying [24], dip coating [25], sputter coating [26], biomimetic coating [27], and electrophoretic deposition are widely investigated [28]. The electrodeposition technique is one of the prominent coating methods because of fabrication at low process temperature, process simplicity, and uniformity of deposition [29,30]. It has been reported that porous HAp coating can be achieved at high current densities between 5 and 9 mA/cm^2^ by the electrodeposition method [31].

The purpose of this bilayer coating was to discover the corrosion protection behavior of an electrodeposition of the coating in simulated body fluid (SBF) solution. Hence the present work was designed in such a way that Mn-HAp coating on ZnO coated 316L SS alloy improved corrosion resistance, mechanical strength, and biological properties. There are no previous reports available on Mn-HAp/ZnO bilayer coating on 316L SS. This is anticipated to be a superior appropriate alternative material for orthopedic implant compared to the existing coating materials.

## 2. Result and Discussion

### 2.1. Field Emission Scanning Electron Microscopy (FE-SEM) Analysis

**Figure 1:**
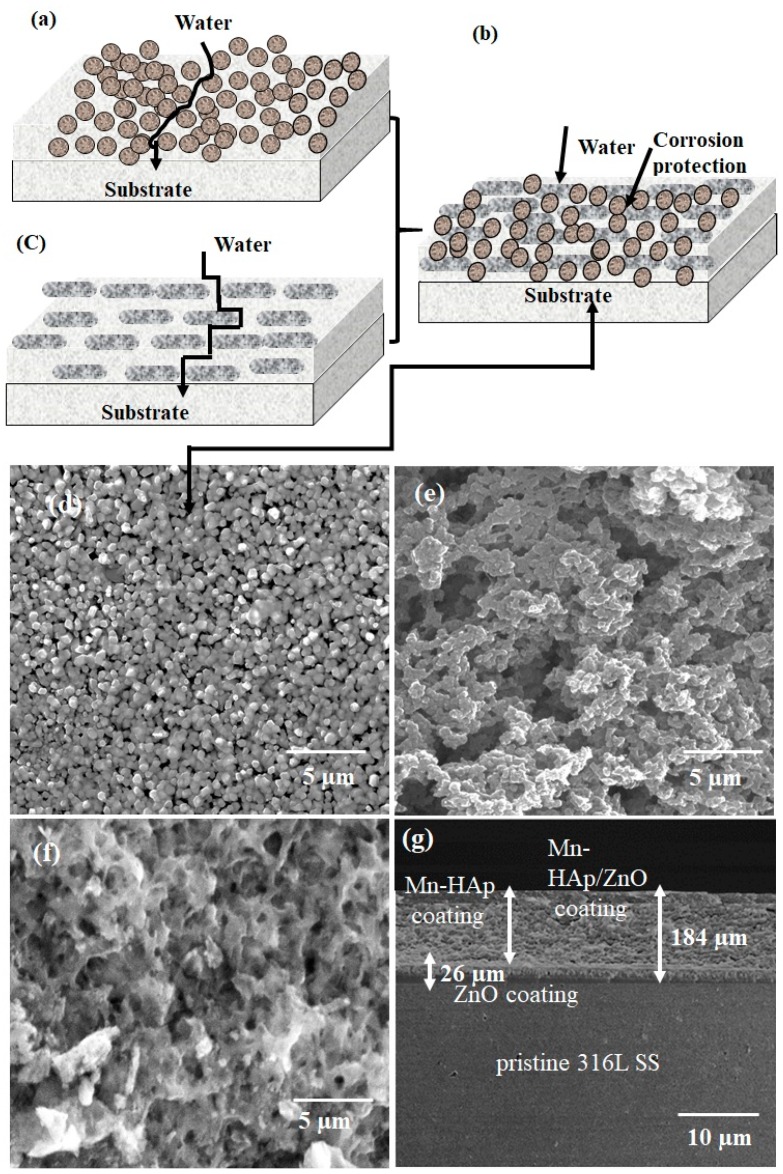
(**a**–**c**) Spherical, lamellar, and combined zinc oxide (ZnO) structure coated stainless steel (316L SS) substrate. Field emission scanning electron microscopy (FESEM) images of (**d**) ZnO, (**e**) porous manganese substituted hydroxyapatite (Mn-HAp), and (**f**) Mn-HAp/ZnO bilayer coatings on 316L SS and (**g**) cross-sectional FESEM image of Mn-HAp/ZnO bilayer coating on 316L SS.

The difference of barriers created by the spherical, lamellar, and combined structure coated on 316L SS substrate model is shown in Figure 1a–c. The developed spherical and lamellar shaped ZnO coatings show a favorable improvement as a barrier against corrosion [32]. Figure 1a–c shows field emission scanning electron microscopy (FESEM) images of ZnO, Mn-HAp, and Mn-HAp/ZnO bilayer coating on 316L SS, and Figure 1d gives cross-sectional images of the bilayer coating. Figure 1d displays the spherical and lamellar shaped ZnO nanoparticles agglomerated with uniformly stiffened grains on 316L SS surface. This small spherical morphology of the particles supports the better anticorrosive coating properties [33,34]. Figure 1e exhibits the formation of the rough microstructure of Mn-HAp on 316L SS. The uniform distribution of the interconnected porous network of Mn-HAp/ZnO is demonstrated in Figure 1f. The interconnected pores, which allow the attachment and proliferation of diverse cell types, are responsible for the formation of functional tissues and support of bone growth [35]. Figure 1g shows a cross-sectional view of the Mn-HAp/ZnO bilayer coating on 316L SS, with a thickness of 184 µm. The cross-sectional layer is continuous without cracks or breaks throughout its length.

### 2.2. Transmission Electron Microscopy (TEM), High-Resolution Transmission Electron Microscopy (HRTEM), and Selected Aread Electron Difraction (SAED) Pattern

**Figure 2:**
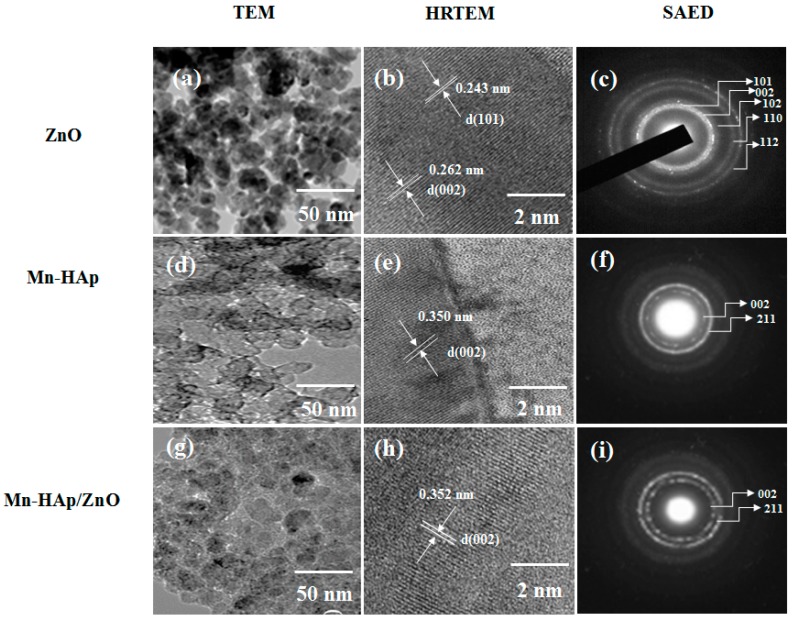
Transmission electron microscopy (TEM), high-resolution TEM (HRTEM), and SAED patterns of (**a**–**c**) ZnO, (**d**–**f**) Mn-HAp and (**g**–**i**) Mn-HAp/ZnO bilayer coating on 316L SS.

Transmission electron microscopy (TEM), high-resolution TEM (HRTEM), and SAED patterns of ZnO, Mn-HAp, and Mn-HAp/ZnO coatings on 316L SS specimens are depicted in Figure 2a–i. Figure 2a shows the spherical and lamellar shaped ZnO merged with the substrate along with small clusters embedded in the substrate. The average size of the ZnO nanoparticles is observed to be ~15 nm. From Figure 2b, the HRTEM image suggests that interplanar distances of 0.262 and 0.243 nm correspond to the (002) and (101) planes of wurtzite ZnO. This is further supported by the formation of a distinguishable ring pattern representing 101, 002, 102, 110 and 112 planes of the SAED pattern (Figure 2c). Figure 2d–f shows the TEM, HRTEM, and SAED patterns of Mn-HAp coating on 316L SS. The TEM image shows uniform agglomeration of porous structure, and the HRTEM exhibits the lattice fringe image in 002 direction, indicating the hexagonal structure of HAp.

The interplanar d spacing value is 0.350 nm, which corresponds to the 002 reflection of a hydroxyapatite phase. The spots corresponding to the (002) faces of Mn-HAp are notably brighter, which suggests an orientation of the Mn-HAp porous structure on the 316L SS substrate. Figure 2g–i shows the Mn-HAp/ZnO bilayer coating on 316L SS substrate. The strong porous coatings are observed in Figure 2g in the TEM images. Figure 2h (HRTEM) indicates the bilayer coatings in virtual settlement with the polycrystalline lattice structure of HAp. However, there is no obvious diffraction in the SAED patterns when compared to the individual coatings of Mn-HAp. Figure 2i shows the SAED images of bilayer coating, and the interplanar spacing (d) is 0.352 nm corresponding to the 002 reflection of apatite phase.

### 2.3. X-ray Diffraction (XRD) Analysis

**Figure 3:**
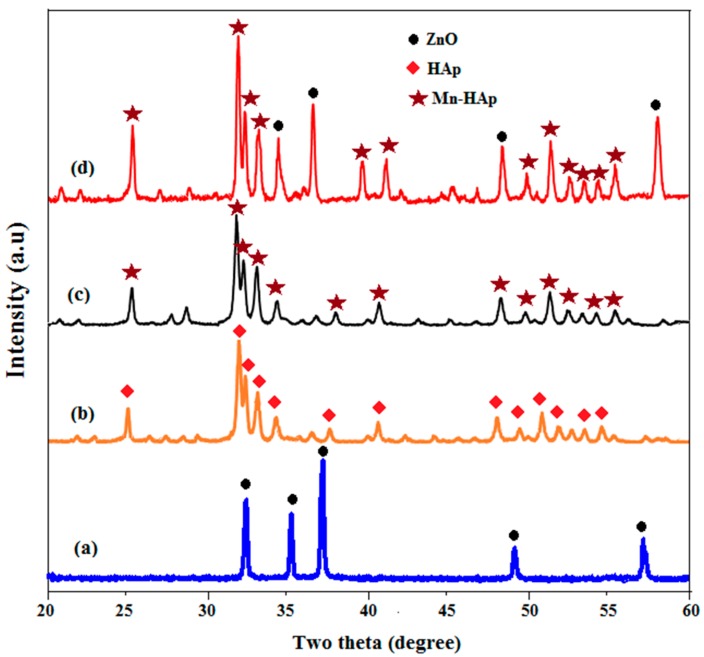
X-ray diffraction (XRD) patterns of (**a**) ZnO, (**b**) HAp, (**c**) Mn-HAp, and (**d**) Mn-HAp/ZnO bilayer coating on 316L SS.

Figure 3a–d shows X-ray diffraction (XRD) patterns for the ZnO, HAp, Mn-HAp, and Mn-HAp/ZnO bilayer coating on 316L SS alloy. From the diffraction pattern in Figure 3a, it can be observed that peak position at 2θ = 33.18°, 35.06°, 37.46°, 49.21° and 52.24° correspond to (110), (002), (101), (102) and (110) reflection planes, respectively. This is in accordance with ZnO wurtzite structure corresponding to standard Joint Committee on Powder Diffraction Standards (JCPDS) (#36-1451).

The sharp reflection peaks indicate good crystallinity of the coated samples. The three high-intensity peaks in Figure 3b, located at 2θ = 31.9°, 32.8° and 32.10°, and the corresponding hkl values (211), (112) and (300) were due to the HAp coating on 316L SS. Figure 3c shows the XRD pattern of Mn-HAp coating on 316L SS alloy. The substitution of Mn ion into the HAp did not affect the diffraction pattern, except some changes in peak width and intensity, indicating the presence of Mn ions in HAp. The XRD pattern of Mn-HAp/ZnO bilayer coating on 316L SS is shown in Figure 3d. The observed intense peak 2θ values of 25.50, 31.41, 33.20, 39.43, 46.82 and 49.68, corresponding to HAp (002), (211), (222), (212), (222) and (213) reflection planes, respectively, were assigned to Mn-HAp, whereas the remaining peaks indicate the (002), (101), (102) and (110) hkl planes, which were assigned to the ZnO of bilayer coating. This combination of two hkl values was confirmed by bilayer coating on 316L SS substrate.

### 2.4. Fourier Transform Infrared Spectroscopy (FT-IR) Analysis

**Figure 4:**
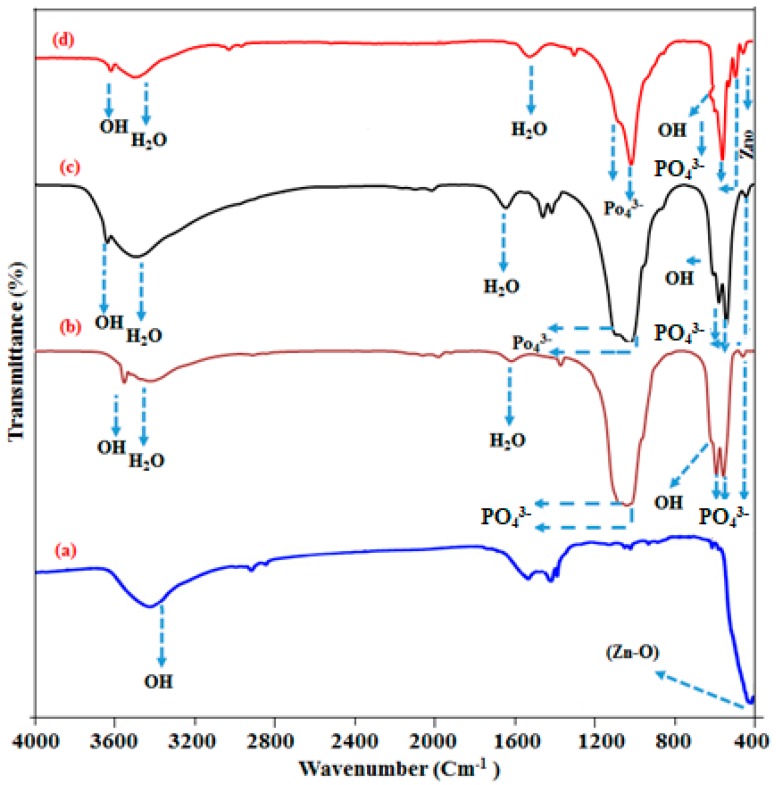
FTIR spectra of (**a**) ZnO, (**b**) HAp, (**c**) Mn-HAp, and (**d**) Mn-HAp/ZnO bilayer coating on 316L SS.

Figure 4a–d shows the Fourier transform infrared spectroscopy analysis of ZnO, HAp, Mn-HAp, and Mn-HAp/ZnO bilayer coating on 316L SS alloy. Figure 4a shows ZnO coating on 316L SS, with the characteristic absorption band at 440 cm^−1^ attributed to ZnO stretching vibration mode [36]. The bond located at 3465 cm^−1^ indicates the stretching vibration of the O–H bond. The weak band near 1512 cm^−1^ is assigned to H–O–H bending vibration. Figure 4b shows FTIR spectrum of HAp coated on 316L SS. The characteristic peaks PO_4_^3−^ were located at 1084 cm^−1^ (v_3_), 596 cm^−1^ (v_4_), and 468 cm^−1^ (v_2_), 946 cm^−1^ (v_1_). The bands situated at 3446 cm^−1^ and 1627 cm^−1^ were due to the stretching and bending mode of adsorbed water (H_2_O) molecules. The bond present at 636 cm^−1^ and 3578 cm^−1^ could be attributed to the bending and stretching vibration of the hydroxyl (O–H) group of HAp [37]. The FTIR spectra of the Mn-HAp samples are shown in Figure 4c. The bands appearing at 3442 cm^−1^ and 1632 cm^−1^ could be due to the presence of stretching and bending mode of water (H_2_O) molecules. The main Mn-HAp characteristic PO_4_^3−^ peaks were observed at 1086 cm^−1^ (v_3_), 594 cm^−1^ (v_4_), 463 cm^−1^ (v_2_), and 945 cm^−1^ (v_1_). The stretching and bending mode of OH was also seen in the spectrum region 3587 and 636 cm^−1^. Figure 4d shows the Mn-HAp/ZnO bilayer coating on 316L SS. All these peaks confirm the presence of Mn-HAp bilayer coating on 316L SS, and some of the ZnO peaks were present in the spectrum, which strongly confirms that the bilayer coating was present in the substrate.

### 2.5. X-ray Photoelectron Spectroscopy (XPS) Analysis

**Figure 5:**
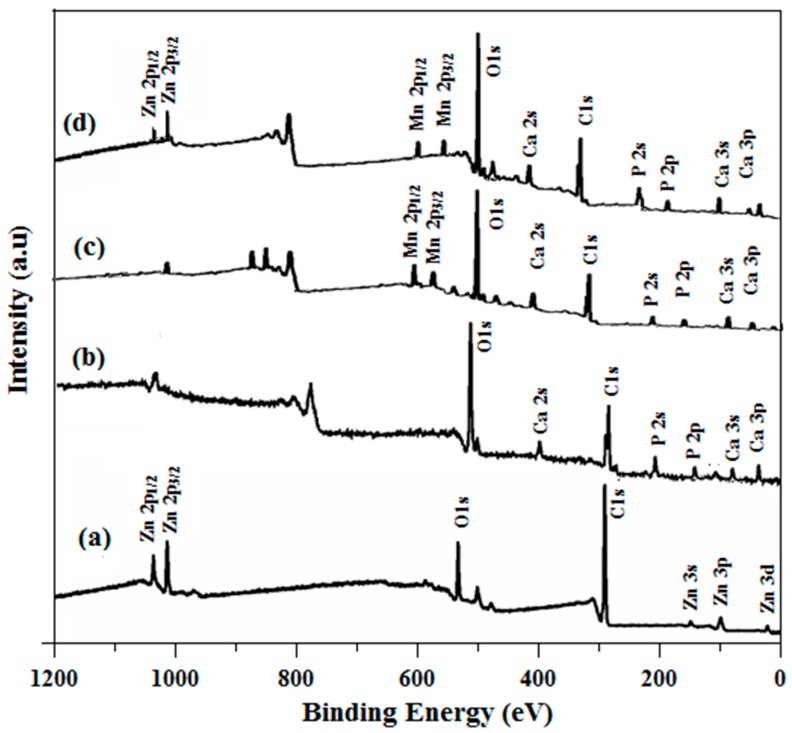
XPS analysis of (**a**) ZnO, (**b**) HAp, (**c**) Mn-HAp, and (**d**) Mn-HAp/ZnO bilayer coating on 316L SS.

**Figure 6:**
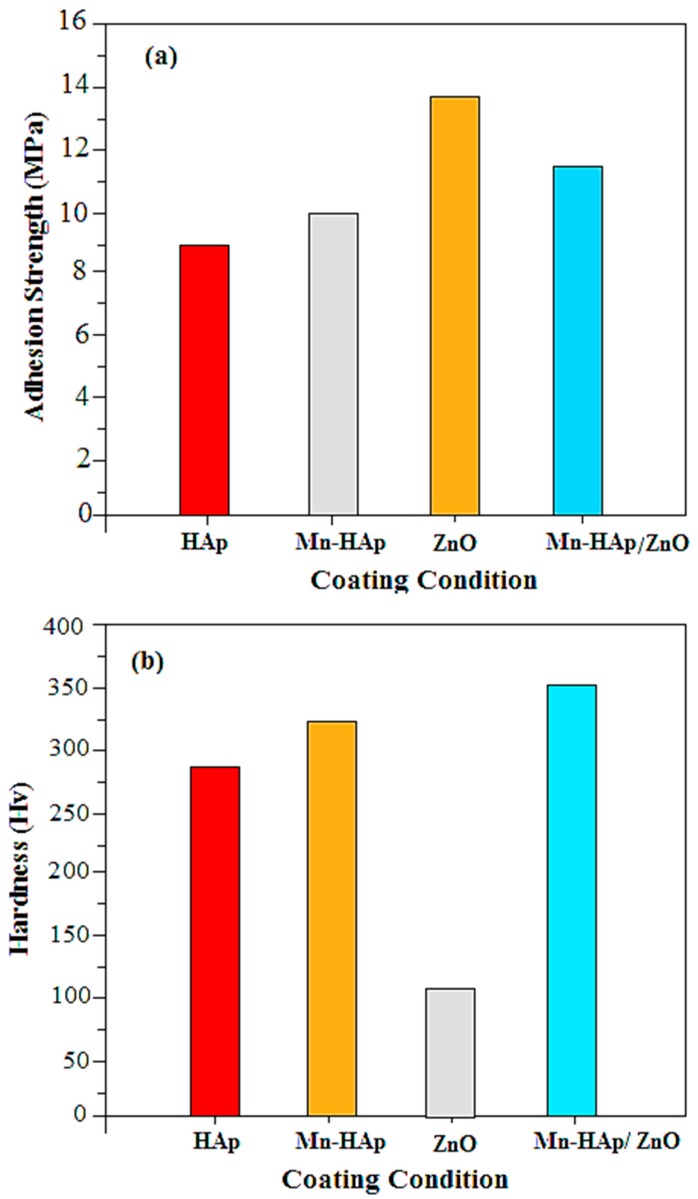
(**a**) Adhesion strength; (**b**) hardness strength of HAp, Mn-HAp, ZnO and Mn-HAp/ZnO bilayer coating on 316L SS.

Figure 5a–d shows the surface composition of ZnO, HAp, Mn-HAp, and Mn-HAp/ZnO bilayer coatings. Figure 5a displays the XPS spectra of ZnO coating on 316L SS. The peak positions at 1022.6 and 1043.7 eV resemble the Zn2p_3/2_ and Zn2p_1/2_ core levels, respectively. The asymmetric peak is observed in the O1s region for ZnO coated samples and the corresponding binding energy is 530.2 eV [38]. The peak at 290.4 eV is attributed to C 1s XPS peak. From the XPS spectrum of HAp coating on 316L SS, as shown in Figure 5b, the elemental position of O1s, Ca2p_1/2_, Ca2p_3/2_, P2p, and C1s peaks corresponds to the binding energies 534.3, 353.2, 351.3, 135.8, and 237.9 eV [39]. From Figure 6c, it can be concluded that there is no major dissimilarity between HAp and Mn-HAp, except that the Mn ions are incorporated into the HAp crystal structure and the binding energies of Mn 2p_1/2_ and Mn 2p_3/2_ are 643.3 and 655.6 eV, respectively. Figure 5d shows the XPS spectrum of Mn-HAp/ZnO bilayer coating on 316L SS. Here most of the Mn-HAp and ZnO peaks are mixed together, with no other impurity peaks, which indicates the strong attachment of Mn-HAp with the bilayer coating on 316L SS.

### 2.6. Mechanical Characterization

The mechanical strength analysis is an important parameter for bioimplants, since it gives information about the load-bearing affinity under stress when the device is implanted into the human body. Figure 6a shows the adhesion strength of the HAp, Mn-HAp, ZnO, and Mn-HAp/ZnO bilayer coatings on 316L SS alloy. The adhesion strength of the pristine HAp and ZnO coating is 8.9 and 13.8 MPa, respectively, while the Mn-HAp and Mn-HAp/ZnO bilayer coating values are 10.2 and 11.6 MPa. The increased adhesion strength of the bilayer coating is due to the unique microstructure of the ZnO-coated 316L SS surface. Figure 6b shows the hardness test of the pristine 316L SS and the Mn-HAp, ZnO, and Mn-HAp/ZnO bilayer coating on 316L SS alloy. For the pristine 316L SS and Mn-HAp and ZnO coating, the Vickers microhardness values are found to be 293 ± 52, 326.7 ± 23 and 96 ± 24, respectively. The Hv value (362 ± 69) obtained for the Mn-HAp/ZnO bilayer coating was higher than that of the other samples. The increased mechanical strength was influenced by mechanical interlocking and chemical bonding, which was improved by sintering. The micron-size particle could improve the mechanical strength and very long-term functionality of the coating [40]. This improved adhesion strength and hardness of the as-formed Mn-HAp/ZnO bilayer coating on 316L SS substrate, making it suitable for biomedical applications.

### 2.7. Electrochemical Characterization of Coating

#### 2.7.1. Potentiodynamic Polarization Measurements

**Figure 7:**
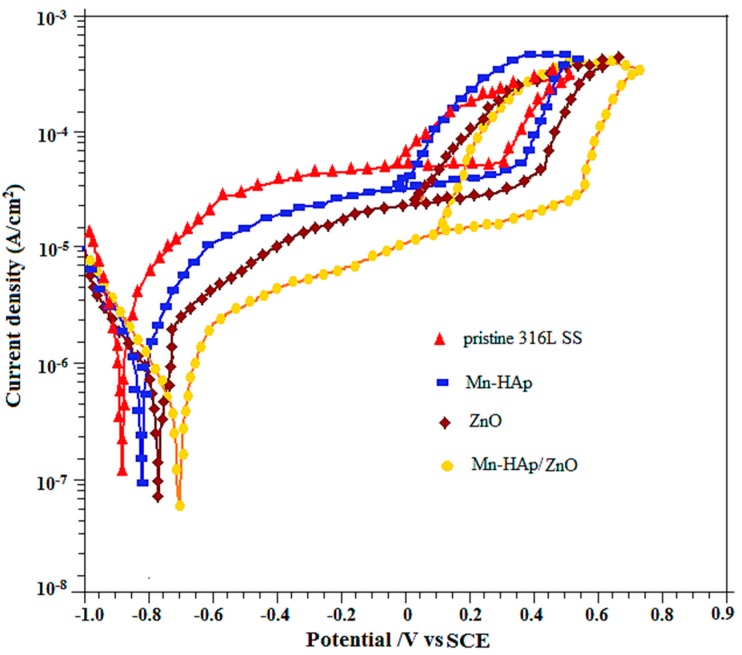
Potentiodynamic cyclic polarization curves of pristine 316L SS, and Mn-HAp, ZnO, and Mn-HAp/ZnO bilayer coating on 316L SS in simulated body fluid (SBF) solution.

The potentiodynamic cyclic polarization curves of pristine 316L SS, and Mn-HAp, ZnO and Mn-HAp/ZnO bilayer coatings on 316L SS in SBF solution were recorded in the potential range of −1.0 V to 0.9 V in order to study the passivation and breakdown behavior of both coating types, and are shown in Figure 7. The corrosion potential (E_corr_), breakdown potential (E_b_), and repassivation potential (E_pp_) curves of the samples were determined from the polarization curve values presented in Table 1. The polarization plots of the Mn-HAp, ZnO and Mn-HAp/ZnO bilayer coatings on 316L SS samples showed a significant shift toward the nobler direction compared to the pristine 316L SS sample. Polarization curves showed that the E_corr_, E_b_ and E_pp_ values for the pristine 316L SS alloy were −874 mV, +348 mV and −75 mV vs. saturated calomel electrode (SCE), respectively. The polarization curve recorded for Mn-HAp coated 316LSS alloy showed E_corr_, E_b_ and E_pp_ values of −832 mV, +410 mV and −48 mV vs. SCE, respectively. The polarization curve of ZnO coated 316L SS alloy showed E_corr_, E_b_ and E_pp_ values of −781 mV, +486 mV and 26 mV vs. SCE, respectively. The shift in E_corr_, E_b_ and E_pp_ values toward the noble direction is an indication that Mn-HAp/ZnO bilayer coating on 316L SS alloy has high corrosion protection in SBF solution. The E_corr_, E_b_ and E_pp_ values for the pristine 316L SS alloy were −696 mV, +574 mV and −92 mV vs. SCE, respectively. The ZnO coating layer offers corrosion protection of metallic substrates by acting as a barrier against electron and ion diffusion, thus dropping the electrochemical reactions at the interface of 316L SS and electrolyte. It also forms a densely filled and crack-free coating. This coating acts as a barrier between the uncoated surface and the SBF solution, enhancing corrosion protection when associated with the individual coating [41].

#### 2.7.2. Electrochemical Impedance Spectroscopy (EIS) Analysis

**Figure 8:**
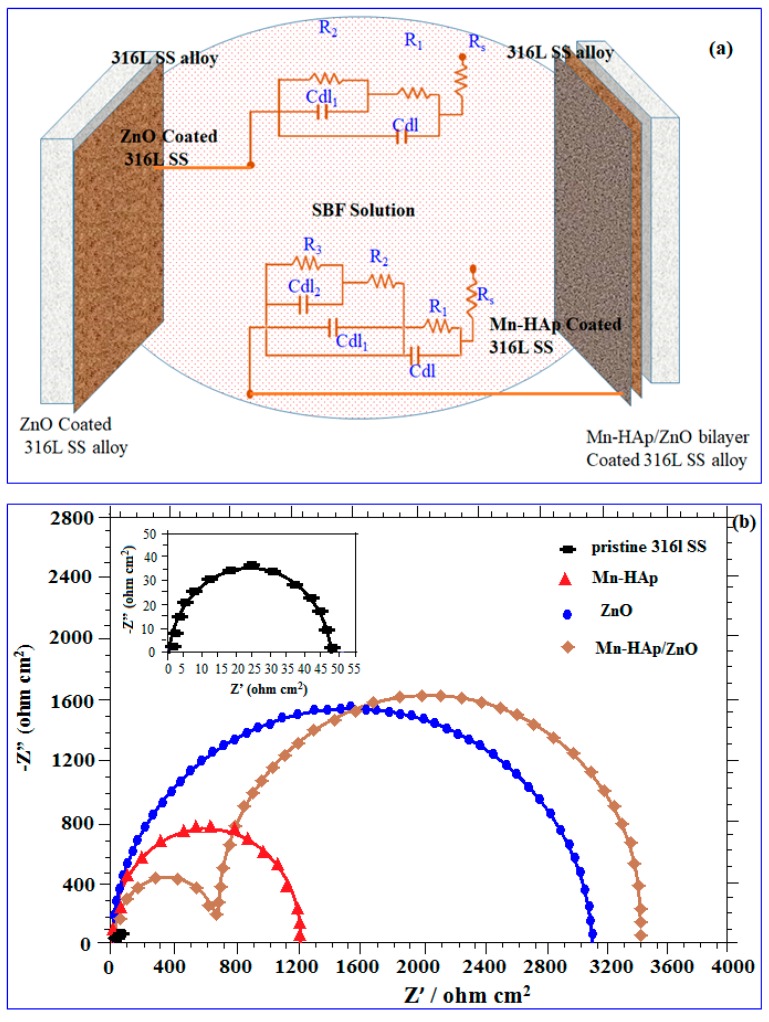
Equivalent circuit obtained for (**a**) ZnO coated and Mn-HAp/ZnO bilayer coated 316L SS; (**b**) potentiodynamic Nyquist plot analysis of pristine 316L SS, and Mn-HAp, ZnO, and Mn-HAp/ZnO bilayer coated 316L SS in SBF solution. C_dl_ represents the first layer capacitance and R_1_ represents resistance to the charge transfer of oxidation for uncoated 316L SS. R_2_ and C_dl1_ represent resistance and capacitance of the ZnO layer, respectively.

An extended electrochemical study was conducted on 316L SS with protective Mn-HAp/ZnO coatings in order to follow its corrosion performance in SBF solution for long-term implant applications. EIS spectra were analyzed with an equivalent circuit and curve fitting was performed for all substrates, showing excellent agreement between the experiments and the fitting. The impedance spectra obtained for pristine, ZnO coated, and Mn-HAp/ZnO coated 316L SS specimens were fitted using an equivalent circuit model, as shown in Figure 8a. The fitted equivalent circuit model, denoted as R_s_ (R_1_C_dl_) (R_2_C_dl_) in Figure 8a, consists of two combinations of resistors and capacitors in series with the solution resistance, used to obtain the spectrum for ZnO coated 316L SS substrate. R_s_ represents the solution resistance or ohmic resistance of the system.

Figure 8a displays the equivalent circuit used to fit the spectrum attained for Mn-HAp/ZnO bilayer coating on 316L SS substrate containing the three combinations of resistor and capacitor in series with solution resistance, represented as (R_2_C_dl1_) (R_3_C_dl2_), where R_3_ and C_dl2_ are resistance and capacitance, respectively. This indicates the presence of two time constants, corresponding to the inner ZnO layer and top Mn-HAp layer. A very large R_p_ is related to a slower rusting system. Moreover, superior corrosion protection delivered by an inhibitor is linked with a reduction in C_dl_ [42]. The reduction in C_dl,_ results from a decrease in local dielectric constant and/or an increase in the thickness of the electrical double layer [43].

The electrochemical impedance spectra in the form of Nyquist plots for the pristine 316L SS, and Mn-HAp, ZnO, and Mn-HAp/ZnO bilayer coating on 316L SS in SBF solution under open circuit potential (OCP) conditions is shown in Figure 8b. The polarization resistance (R_b_) value for the pristine, Mn-HAp, and ZnO coated 316L SS was 48, 1200 and 2980 Ω/cm^2^, respectively. The maximum R_b_ 3400 Ω/cm^2^ value was obtained for Mn-HAp/ZnO bilayer coating, showing more corrosion protection than other samples. Two capacitive semicircles were obtained for the Mn-HAp/ZnO bilayer coating; the first, at higher frequencies, can be attributed to the ZnO layer and the second, at low frequencies, is attributed to the compact Mn-HAp layer. This result is typically witnessed for bilayer coatings containing a dense interior (bottom) layer and a less compact (porous) exterior layer. Considering this, an equivalent circuit was created to simulate the results. In all spectra, the entire value of the maximum phase angle was less than 90° [44]. From these results, enhancement of the corrosion protection of the bilayer coating is most suitable for implant applications.

### 2.8. In Vitro Bioactivity

**Figure 9:**
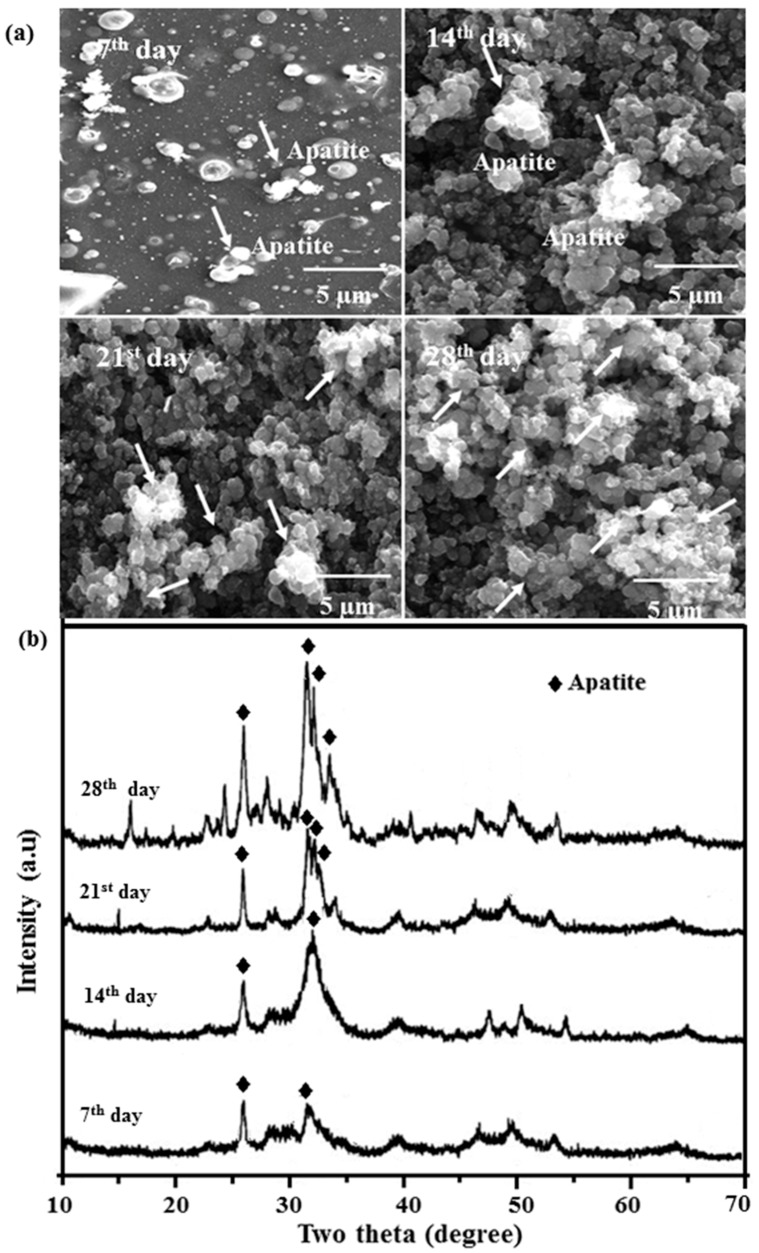
(**a**) FESEM images and (**b**) XRD pattern of Mn-HAp/ZnO bilayer coating on 316L SS at 7, 14, 21 and 28 days of incubation in SBF solution.

Figure 9a shows FESEM images of the apatite formation of Mn-HAp/ZnO bilayer coating on 316L SS after immersing in SBF for 7, 14, 21 and 28 days. After 7 days, a single layer of spherical particles started to deposit on the surface. After 14 days, apatite deposition was rapidly enriched, and after 21 days the surface was covered with particles. Increasing surface nonuniformity was seen due to the increasing Ca-P depositions on the surface. With a further increase in soaking time of 28 days, minor clusters of apatite were detected completely covering the bilayer coating. During the incubation period, calcium (Ca) ions from SBF were attracted by OH^−^ and the exchange of Ca^2+^ and H^+^ with OH^−^ resulted in higher pH. Accumulation of OH^−^ on the surface is necessary for apatite nucleation [45]. As Ca and phosphate (P) ion concentrations reached their maximum, pH also increased. Ca and P ions are consumed largely due to the formation of abundant apatite. Since there is a corresponding consumption of OH^−^, the pH of SBF decreases. When the equilibrium of dissolution and precipitation is achieved, pH becomes constant. Normally HAp dissolution occurs in five steps as follows:

(1) $${\mathrm{Ca}}_{5}{\left({\mathrm{PO}}_{4}\right)}_{3}{\mathrm{OH}}_{\left(s\right)}+{H^{+}}_{\left(\mathrm{aq}\right)}⇄{\mathrm{Ca}}_{5}{{(\mathrm{PO}}_{4})}_{3}{{{(H}_{2}O)}^{+}}_{\left(s\right)}$$

(2) $${2\mathrm{Ca}}_{5}{\left({\mathrm{PO}}_{4}\right)}_{3}{{{(H}_{2}O)}^{+}}_{\left(s\right)}⇄3{\mathrm{Ca}}_{3}{{(\mathrm{PO}}_{4})}_{2(s)}+{{\mathrm{Ca}}^{2+}}_{\left(\mathrm{aq}\right)}+2H_{2}O$$

(3) $${\mathrm{Ca}}_{3}{{(\mathrm{PO}}_{4})}_{2(s)}+2{H^{+}}_{\left(\mathrm{aq}\right)}⇄{{\mathrm{Ca}}^{2+}}_{\left(\mathrm{aq}\right)}+2{\mathrm{CaHPO}}_{4(s)}$$

(4) $${\mathrm{CaHPO}}_{4(s)}+{H^{+}}_{\left(\mathrm{aq}\right)}⇄{{\mathrm{Ca}}^{2+}}_{\left(\mathrm{aq}\right)}+H_{2}{\mathrm{PO}}_{4(\mathrm{aq})}^{-}$$

(5) $${\mathrm{CaHPO}}_{4(s)}⇄{{\mathrm{Ca}}^{2+}}_{\left(\mathrm{aq}\right)}+{\mathrm{HPO}}_{4(\mathrm{aq})}^{2-}$$

Figure 9b shows the apatite formation ability of Mn-HAp/ZnO bilayer coating on 316L SS samples soaked in SBF solution at 7, 14, 21 and 28 days. Two low-intensity apatite peaks 2θ = 26.14° and another peak between 31.24° and 33.06° were first observed at 7 days of immersion, with the wide-ranging apatite peaks designated low crystallinity of apatite formed in vitro at this initial stage of soaking. The HAp triplet peak and ZnO peaks appeared in the Mn-HAp/ZnO bilayer coating before immersion in SBF (Figure 3d). Now the peaks were suppressed at 7 and 14 days of incubation (Figure 9b), suggesting that surface chemistry processes were occurring in the samples. The intensity of apatite peaks increased gradually with immersion time, indicating the growth of an apatite layer on the composite surface in SBF, and another two peaks at 33.02° and 35.14° appeared after 14 and 28 days of immersion. The rapid increase of apatite peak intensity indicates more biomineralization ability in the present coating.

### 2.9. Inductively Coupled Plasma Atomic Emission Spectrometry (ICP-AES) Analysis

**Figure 10:**
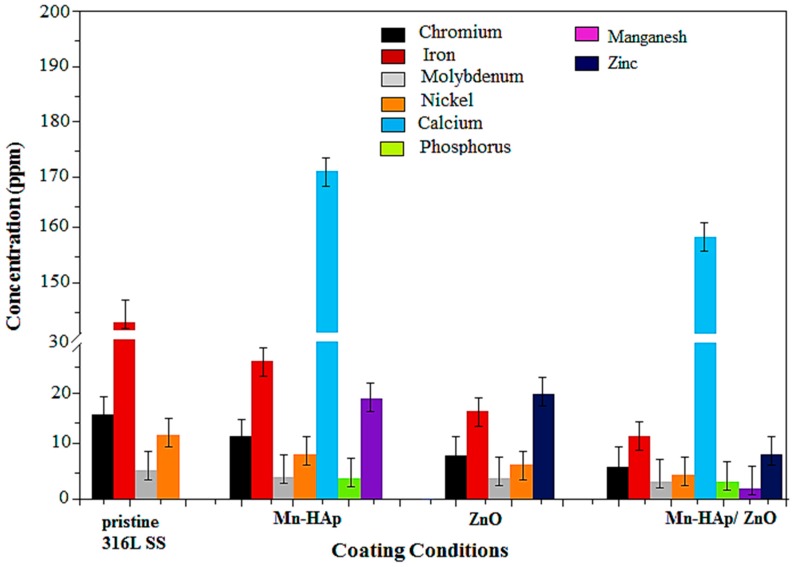
Leach-out analysis of pristine 316L SS, and Mn-HAp, ZnO, and Mn-HAp/ZnO bilayer coating on 316L SS. [The % error of the measured sample associated to the true value is determined by the following equation: % error = Absolute value [(true value-measured value)/true value] × 100. Acceptance criteria is a %RSD and a % Error less than 10%.].

Potentiodynamic polarization tests were performed at a constant potential of 0.45 mV vs. SCE after aging of 1 h to determine the concentration of metal atoms, namely chromium (Cr), nickel (Ni), molybdenum (Mo), and iron (Fe), which are leached out from the 316L SS during the corrosion process. These results specify the resistance of the alloys to the toxic metal ions, which produce local systemic effects and thereby play a crucial role in prosthetic loosening. The rate of surface dissolution can be identified by monitoring the evolution of ions from the implant material into the solution. Figure 10 shows the concentration of leached out metal ions from the pristine 316L SS, and Mn-HAp, ZnO, and Mn-HAp/ZnO bilayer coating on 316L SS. A substantial amount of Fe, Cr, Ni, and Mo atoms were leached out from the pristine 316L SS [46]. This indicates that no barrier film on the 316L SS surface prevents the attack of chloride ions in the SBF solution. However, the rate of dissolution of ions has little control on Mn-HAp coated on 316L SS, only a considerable amount of manganese and phosphate are released from the coated substrate. The ZnO coating on 316L SS substrate exposes the amount of leached out metal ions, which was lower than that of the pristine 316L SS and Mn-HAp coating. Furthermore, the leach-out is extremely reduced in Mn-HAp/ZnO bilayer coating and it prevents the unwanted metal ions from going into the body. Hence the prepared bilayer coating is highly suitable for bioimplants.

### 2.10. In Vitro Biocompatibility Cell Culture Studies

#### 2.10.1. Cell Viability

**Figure 11:**
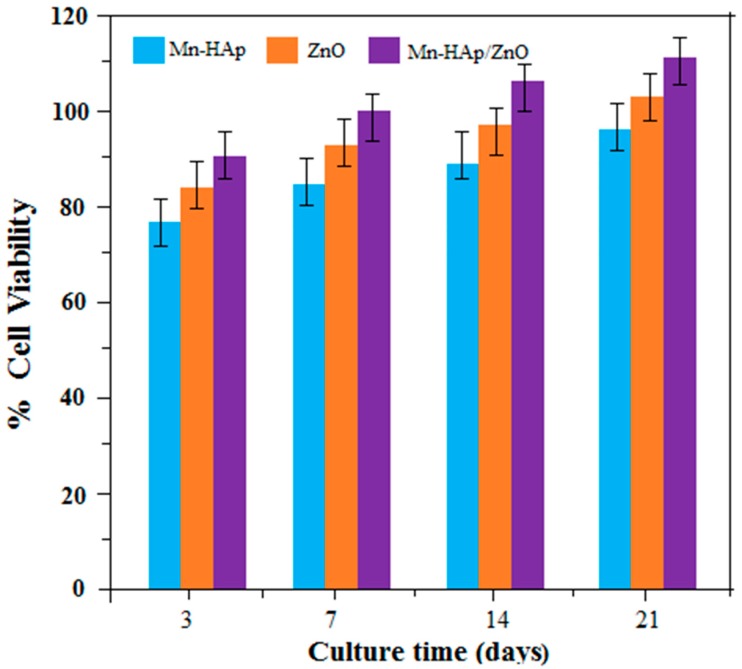
Cell viability of Mn-HAp, ZnO, and Mn-HAp/ZnO bilayer coating on 316L SS.

The enhanced cell viability of the bilayer coating is mainly due to the presence of porous Mn-HAp coating on ZnO coated 316L SS. Cell viability of 125 µg mL^−1^ of Mn-HAp, ZnO, and Mn-HAp/ZnO bilayer coating on 316L SS substrates at 3, 7, 14, 21 days of culture is shown in Figure 11. Cell viability in the coated samples was studied using the MTT (3-(4,5-dimethylthiazol-2-yl)-2,5-diphenyl tetrazoliumbromide) assay, which is a measure of the mitochondrial activity in cells. The results show that cell viability of the bilayer coating (85%, 95%, 102% and 108%) increased extensively compared to the Mn-HAp coating (73%, 81%, 84% and 87%) and ZnO coating (82%, 89%, 92% and 96%) at 3, 7, 14 and 21 days of culture, respectively. Statistical analysis was carried out on cellular tests using one-way analysis of variance (ANOVA) at an average of 3–5 replicates. Differences were considered statistically significant at *p* < 0.05. These results suggest that the bilayer coating shows the best cell viability and so is highly suited for biomedical applications.

#### 2.10.2. Cell Adhesion

**Figure 12:**
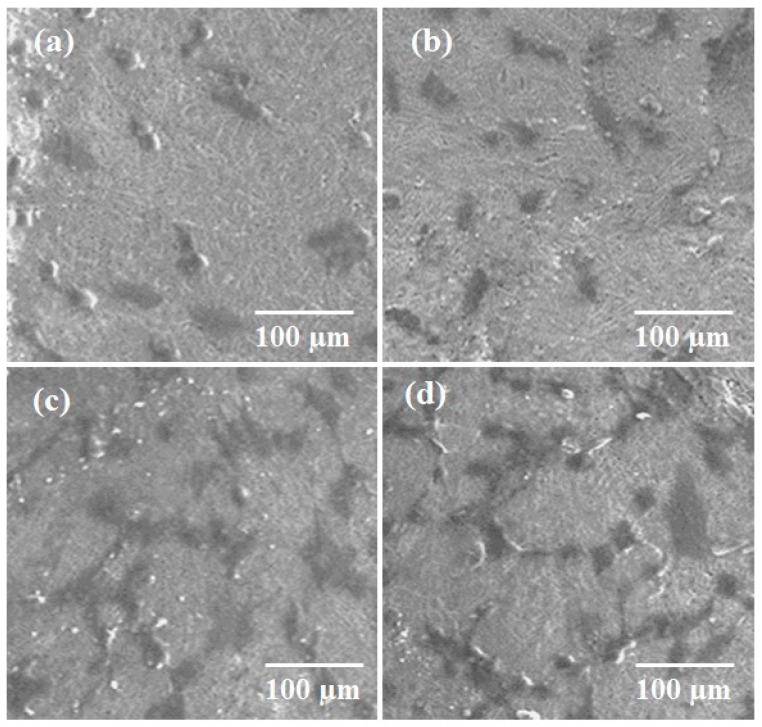
(**a**–**d**) FESEM images of MG-63 osteoblast cell proliferation of Mn-HAp/ZnO bilayer coating on 316L SS substrate at (**a**) 3, (**b**) 7, (**c**) 14 and (**d**) 21 days.

Adhesion strength is an important property for in vivo implantation. Here the adhesion strength of Mn-HAp/ZnO bilayer coating on 316L SS alloy was evaluated at 3, 7, 14 and 21 days. After 3 and 7 days of culture, the cells were attached on the surfaces and exhibited a heterogeneous morphology spreading on the substrate (Figure 12a,b). Calcium and phosphorous are also vital in improving osteoblastic cell behavior and in vivo bone regeneration. Ca^2+^ has a crucial role in bone regeneration and controls the proliferation and differentiation of target osteoblasts [47]. After 14 days of culture, the osteoblast cells were elongated with only limited areas of spreading at the ends of long filopodia structures, as shown in Figure 12c. Finally, after 21 days of culture, there were cell-to-cell contacts and polygonal or bipolar morphology with extensions in various directions on the surface coatings, shown in Figure 12d. The porous structured layer of bilayer coating offers enhanced bioactivity due to surface roughness, which leads to increased surface energy [48].

#### 2.10.3. Live/Dead Assay

**Figure 13:**
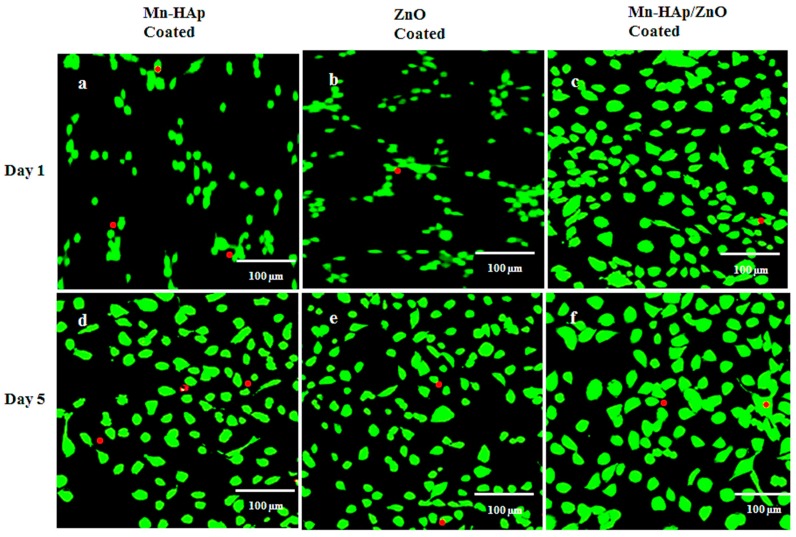
(**a**–**f**) Live/dead cell analysis of (**a**,**d**) Mn-HAp, (**b**,**e**) ZnO, and (**c**,**f**) Mn-HAp/ZnO bilayer coating on 316L SS at 1 and 5 days of culture. Red color—dead cells, Green Color—Live Cells.

Cytocompatibility analysis of Mn-HAp, ZnO, and Mn-HAp/ZnO bilayer coating on 316L SS was performed to assess live/dead staining, depicted in Figure 13. In the images of live/dead staining, viable cells are shown in green and dead cells in red. Visual inspection readily shows sustained attachment of the coatings and growth of specific osteoblast cell types, supporting the overall biocompatibility of the coatings. All the groups showed increased cell proliferation on the fifth day when compared to the first day. Figure 13c,f shows the live/dead staining of Mn-HAp/ZnO bilayer coating, which had the least dead cells and most living cells compared to Mn-HAp (Figure 13a,d) and ZnO (Figure 13b,e). The results of the live/dead assay show that most MG63 cells on all 316 LSS substrates were live (green) except one or two dead cells (red), so these coatings are safe and appropriate for the growth of osteoblasts. The results of the cell culture experiments confirm that the bilayer coating on 316L SS plays a vital role in implant applications.

## 3. Materials and Methods

### 3.1. Materials

Zinc nitrate hexahydrate (Zn(NO_3_)_2_·6H_2_O), calcium nitrate tetrahydrate (Ca(NO_3_)_2_·4H_2_O), diammonium hydrogen phosphate (NH_4_)_2_HPO_4_, manganese nitrate tetrahydrate (Mn(NO_3_)_2_·4H_2_O), ethanol (EtOH), and ammonium hydroxide (NH_4_OH) were purchased from Sigma Aldrich Chemical & Co. (Tamil Nadu, India). All the chemicals were of analytical grade, and deionized water was used throughout the experiment.

### 3.2. ZnO Coating on 316L SS

A conventional cell with a 3-electrode configuration was used for electrochemical deposition, by using an electrochemical workstation (CHI 760C, CH Instruments, Austin, TX, USA) in which platinum electrode was used as the counterelectrode, 316L SS alloy as the working electrode, and saturated calomel electrode (SCE) as the reference electrode. The electrolyte was prepared by dissolving 0.1 M Zn(NO_3_)_2_·6H_2_O into 100 mL of absolute ethanol in a sealed container and stirring continuously for 30 min at room temperature to form a transparent solution, which acted as a precursor [49]. Electrodeposition of ZnO was carried out at room temperature and at a current density of 0.75 mA·cm^–2^ for 60 min. After the deposition of ZnO, the coated 316L SS surface was washed with deionized water to remove residual electrolyte, and after that the coated samples were naturally dried for 24 h.

### 3.3. Mn-HAp Coating on 316L SS

Mn-HAp deposition on 316L SS was carried out in an aqueous solution containing 0.5 M (Ca(NO_3_)_2_·4H_2_O), 0.03 M (NH_4_)_2_·HPO_4_ and 0.003 M (Mn(NO_3_)_2_·4H_2_O) under magnetic stirring at room temperature with the following parameters: pH 4.5, current density 9 mA/cm^2^, and duration 30 min [50]. The coated substrate was gently rinsed with deionized water and then dried at room temperature for 24 h.

### 3.4. Mn-HAp/ZnO Bilayer Coating on 316L SS

Mn-HAp was coated galvanostatically on the ZnO coated 316L SS at a constant current density of 9 mA/cm^2^ for a duration of 30 min. After deposition of the Mn-HAp/ZnO bilayer coating on 316L SS surface, it was washed with deionized water to remove residual electrolyte and dried for 24 h. All potentials in this experiment are quoted on the SCE scale.

### 3.5. Surface Characterization of Coating

The functional group analysis of coating samples was characterized by Fourier transform infrared spectroscopy (FTIR) using a Nicolet 8600 FTIR spectrometer (Tamil Nadu, India). FTIR spectra were recorded from 400 to 4000 cm^−1^ with 4 cm^−1^ resolution, averaging 100 scans. The phase composition of the coatings was analyzed by X-ray diffraction (XRD) using a Bruker D8 Advanced diffractometer (Karlsruhe, Germany) with Cu-Kα radiation, 40 kV/40 mA, and λ = 1.5406 nm. The surface morphology of coating specimens was studied by field emission scanning electron microscopy (FESEM; Quanta 250 FEG, FEI Company, (Hillsboro, OR, USA) at 30 kV. Energy dispersive X-ray Analysis (X Flash Detector, 5030 Bruker Nano, Karlsruhe, Germany) examined the elemental compositions of all coated specimen. The elemental compositions of the present samples were determined by X-ray photoemission spectroscopy (XPS) with a VGS ESCALAB 210 instrument (Oakville, ON, Canada). The inner surface morphology of coatings was obtained by transmission electron microscopy (TEM; JEOL JEM2010, 200 Kv, Pleasanton, CA, USA) and HRTEM (JEM-2100F, Williamston, SC, USA).

### 3.6. Mechanical Properties of Coatings

The mechanical properties of the coatings were analyzed using a Universal Instron Mechanical Testing system (Instron 5565, Instron Co., Norwood, MA, USA) according to ASTM F 1044-05 standards [51], assessing the adhesion strength between HAp, Mn-HAp, ZnO, and Mn-HAp/ZnO bilayer coatings on 316L SS specimens. Adhesion strength is an essential property for in vivo implantation. Six parallel score lines were made 1.0 mm apart; an additional 6 score lines were inscribed vertical to the original lines. For each distinct specimen, 25 grids were produced. Adhesive tape was placed on the grids using a soft eraser; the tape was then detached with a firm and stable pulling action. Hardness of the coatings was determined using Akashi AAV-500 series hardness tester (Kanagawa, Japan). The loading force of 50 g for a duration of 5 s and the hardness measurement were carried out 10 times for coated and uncoated substrates.

### 3.7. Inductively Coupled Plasma Atomic Emission Spectrometry (ICP-AES)

The leached out metal ions from pristine 316L SS, and Mn-HAp, ZnO, and Mn-HAp/ZnO bilayer coating on 316L SS substrate were determined by applying an impressed potential of 455 mv vs. SCE just above the breakdown potential (E_b_) of the pristine 316L SS for 1 h in SBF solution after completion of the potentiodynamic polarization analysis. At the end of each experiment an aliquot of 10 mL of medium was collected for ICP-AES analysis (Thermo Jarrel-Ash Atom scan, Franklin, MA, USA).

### 3.8. Electrochemical Investigation of Coatings

Corrosion performance of pristine 316L SS, and Mn-HAp, ZnO, and Mn-HAp/ZnO bilayer coating on 316L SS alloy was analyzed by potentiodynamic polarization and electrochemical impedance spectroscopy (EIS) in SBF solution. pH and temperature were maintained at 7.4 and 37 °C, respectively. All electrochemical measurements were carried out using the 3 electrode electrochemical workstation with CHI 760 (Austin, TX, USA), The saturated calomel electrode (SCE) and platinum electrode were taken as the reference and counterelectrode, respectively, and coated 316L SS as a working electrode was used for all measurements. All potential values are related to the SCE. Potentiodynamic polarization studies were measured at a scan rate of 1 mV·s^−1^ in the potential range between −1 and 0.9 mV. The breakdown or pitting potential was attained at the potential where there was a monotonic rise in the current density. The repassivation potential (E_pp_) is the potential at which the reverse scan comes across the passive region. Electrochemical impedance studies were done in the same setup as potentiodynamic polarization studies and the applied ac perturbation signal was about 5 mV within the frequency range 10^−2^ Hz–100 kHz. All impedance measurements were carried out under open circuit potential (OCP) conditions. The best achieved data were taken using internally existing software. Individual electrochemical experiments were repeated 3 times to confirm the reproducibility.

### 3.9. Simulated Body Fluid (SBF) Solution Preparation

The standard SBF solution was prepared according to Kokubo’s protocol [52] with NaCl, NaHCO_3_, KCl, K_2_HPO_4_·H_2_O, MgCl_2_·6H_2_O, CaCl_2_, HCl (1 M), Na_2_SO_4_, and NH_2_C (CH_2_OH)_3_. The reagents were dissolved into double distilled water, and 1 M HCl was used to maintain pH at 7.4 at 37 °C. The inorganic ion concentrations in the standard SBF solution are almost the same as in human blood plasma. The bioactivity test was carried out by soaking the coated samples attached vertically in a special platinum holder in 45 mL of SBF in a polyethylene vessel maintained at 37 °C for 7, 14, 21 and 28 days. The SBF solution was renewed every day in order to preserve the ion concentration.

### 3.10. In Vitro Biocompatibility Analysis

#### 3.10.1. Cell Cultures

Human osteoblast-like MG-63 cells were purchased from NCCS, Pune, India. The cultured MG-63 cells in 25 cm^2^ cell culture flask at 37 °C in Dulbecco’s Modified Eagle Medium (DMEM) were combined with 10% fetal bovine serum (FBS) and incubated at 37 °C in 5% CO_2_ for 3, 7, 14 and 21 days. The seeded cells were incubated overnight to allow cell adherence, again at 37 °C in 5% CO_2_ atmosphere, and further used for cell viability studies on the Mn-HAp, ZnO and Mn-HAp/ZnO bilayer coated 316L SS substrates.

#### 3.10.2. Cell Viability

The coating substrates were placed on the attached MG-63 cells and the number of cells was assessed by MTT (3-(4,5-dimethylthiazol-2-yl)-2,5-diphenyl tetrazoliumbromide) assay on cells incubated at 37 °C in 5% CO_2_ for 3, 7, 14 and 21 days. All coated samples were washed using sterilized phosphate buffered saline (PBS). MG-63 cells were seeded in 12-well plates at 10^4^ cells/mL in a humidified 5% CO_2_ atmosphere. After 48 h of incubation, MTT solution in 1 mL serum-free medium was added and incubated with 5% CO_2_, at 37 °C. The data were reported separately for each well by an ELISA reader (Spectra Max 190-microplate reader, Molecular Devices, Delhi, India) at absorbance wavelength 570 nm.

#### 3.10.3. Cell Adhesion Test

Mn-HAp, ZnO, Mn-HAp/ZnO bilayer coatings on the 316L SS samples at 10 days of osteoblast-like cells (1 × 10^5^/cm^2^) were added on the surface of the specimens and incubated in DMEM supplemented with 10% FBS at 37 °C under standard culture conditions. The coating samples were fixed with 2.5% glutaraldehyde in 0.1 M PBS buffer for 20 min and washed 3 times with PBS for 5 min at room temperature. Consequently, each sample was subjected to graded dehydration with pure ethyl alcohol for 10 min at room temperature. The final samples were gold sputter coated for FESEM analysis.

#### 3.10.4. Live/Dead Assay

MG-63 osteoblasts were added in 2 mL of culture medium. MG-63 cells were detached from the culture plate using trypsin/EDTA. The plate was incubated at 37 °C and 5% CO_2_ in the dark for 1 and 5 days. Live/dead stain was prepared by adding 2 µmol/L acetomethoxy derivate of calcein (calcein-AM) and 2 µmol/L ethidium homodimer-1 per milliliter of media. The construct was then left in the incubator for 30 min; afterward, the dye was removed and replaced with 1 mL of DMEM. The ratio of live to dead cells was determined by calculating the number of cells in 3 fields at equal magnification for each Mn-HAp, ZnO, and Mn-HAp/ZnO bilayer coating on 316L SS alloy.

## 4. Conclusions

Bioactive Mn-HAp/ZnO bilayer coating on 316L SS was successfully developed by electrodeposition. The synthesized bilayer coating has improved corrosion resistance, mechanical properties, metal ion leach-out performance, and in vitro bioactivity and biocompatibility. The surface morphology of the bilayer coating has a uniform porous structure and strong adherent coating onto the 316L SS surface. The electrochemical results confirm that the bilayer coating displays excellent corrosion protection when compared to individual coatings in SBF solution. The presence of ZnO coating improves the mechanical strength, whereas the leach-out analysis showed a reduced rate of metal ion dissolution. Moreover, the in vitro bioactivity results indicate well-defined apatite growth. The coating on 316L SS implants shows high cell attachment and proliferation of Mn-HAp/ZnO bilayer coatings. These are the potential materials for bone repair and regeneration.

## Figures and Tables

**Table 1:** Potentiodynamic polarization parameters of pristine 316L SS, and Mn-HAp, ZnO, and Mn-HAp/ZnO bilayer coating on 316L SS. SCE, saturated calomel electrode.

Polarization Parameter				
Sample Condition	E_corr_ (mV vs. SCE)	E_b_ (mV)	E_pp_ (mV)	R_b_ (Ω/cm^2^)
Pristine 316L SS	−874	348	−75	48
Mn-HAp	−832	410	−48	1200
ZnO	−781	486	−26	2980
Mn-HAp/ZnO	−696	574	−92	3400
